# Expansion of Dolomitic Rocks in TMAH and NaOH Solutions and Its Root Causes

**DOI:** 10.3390/ma13020308

**Published:** 2020-01-09

**Authors:** Huan Yuan, Min Deng, Bi Chen, Weifeng Chen, Zhongyang Mao

**Affiliations:** College of Materials Science and Engineering, Nanjing Tech University, Nanjing 210009, China; 201761100162@njtech.edu.cn (H.Y.); xiaochen1123@Yeah.net (B.C.); chenweifeng@njtech.edu.cn (W.C.); mzy@njtech.edu.cn (Z.M.)

**Keywords:** alkali carbonate reaction (ACR), dolomitization, TMAH, ASRgel, expansion, crack

## Abstract

In this paper, a tetramethylammonium hydroxide (TMAH) solution and homemade cement without alkali were used to eliminate the influence of the alkali-silica reaction (ASR) on the expansion of dolomitic rocks, and a NaOH solution was used as a comparison agent. The expansion of concrete microbars and dolomite powder compacts prepared from dolomitic rocks was tested. The expansion cracks and reaction products were investigated by X-ray diffraction, optical microscopy, scanning electron microscopy (SEM) and energy dispersive spectrometry (EDS). The results showed that TMAH reacts with dolomite crystals in dolomitic rocks to form brucite and calcite. Through X-ray diffraction and SEM-EDS analysis, it can be determined that the chemical reaction between TMAH and dolomite crystal was dedolomitization. The expansion stress test and concrete microbar expansion test suggest that the alkali carbonate reaction (ACR) can produce expansion. Although both the ASR and the ACR were observed in the NaOH reaction system, but ASRgel was not found in the cracks, indicating that the ASR may be involved in the expansion process of concrete microbars and that the ACR is the root cause of the expansion. However, under the curing conditions of the TMAH solution, many ACR products were found around the crack, indicating that the expansion of the concrete in this system was caused entirely by the ACR.

## 1. Introduction

In 1940, Stanton [1] first discovered that the alkali-aggregate reaction (AAR) caused damage to concrete. At that time, the term AAR only described the alkali-silica reaction (ASR). In 1950, Swenson [2] proposed a new alkali aggregate reaction called the alkali carbonate reaction (ACR) to explain concrete deterioration. In 1961, Hadley [3] also discovered damage to concrete caused by the ACR in the United States. Subsequently, Deng et al. [4] found that airport pavement in China was destroyed by the ACR, which led to research on the ACR by scholars from various countries. It is generally accepted that the ACR is essentially a chemical reaction between alkali groups in a pore solution and dolomite in an aggregate [5,6] so the ACR is also known as the alkali-dolomite reaction (ADR). However, ACR expansion has been a controversial issue. As early as 1969, Gillott [6] proposed a mechanism by which clay water absorption caused expansion. He believed that ACR expansion was caused by the water absorption and swelling of newly exposed clay and that dolomitization only provided channels for clay water absorption. On the other hand, Tang et al. [7,8,9] believed that brucite and calcite generated by the ADR crystallized and grew in a limited space to generate an expansive force. In addition, researchers also found that there is a material similar to ASRgel in concrete prepared with dolomitic aggregates, and concluded that ACR expansion was caused by the ASR [10]. For example, Katayama [11,12] believed that pure ACR does not cause expansion but that when expansion occurs in carbonate aggregates, it is due to additional components in the rock, such as clay components or microcrystalline quartz, that can undergo the ASR. This finding was also confirmed by Grattan-Bellew [13]. Moreover, Xu et al. [14] observed expansion in carbonate aggregates containing clays. Qian et al. [15] also believed that the ACR plays a key role in the expansion of carbonate aggregates. Milanesi et al. [16,17] used South African dolomite to prepare mortar for rock activity detection. The ACR products brucite and calcite were found in the cracks of the test samples, but ASRgel was not found. The above results indicate that researchers are still divided on whether the ACR can cause expansion and on the causes of this expansion.

According to Thong [18], tetramethylammonium hydroxide (TMAH) has been widely used in silicon anisotropic etching due to the reliable etching of silicon in the fabrication of microelectromechanical systems. During the etching process, TMAH and SiO_2_ serve as the reactant and product, respectively, in the reaction system, so it can be inferred that TMAH does not react with SiO_2_ because the reactant and the reaction product do not react. Chen et al. [19] also reported that ASR reactive components (such as microcrystalline quartz) in dolomitic rocks do not react with TMAH, whereas dolomite in dolomitic rocks may react with TMAH. Therefore, TMAH as a curing solution can effectively eliminate the influence of microcrystalline quartz on dolomitic rock expansion. It is necessary to investigate the reaction behaviour and expansibility of dolomitic rocks in a TMAH solution to determine whether the ACR can cause expansion, and compare these expansion features with those of a sample cured in NaOH solution to clarify the cause of expansion.

In this study, the reaction behaviour of dolomitic rocks in TMAH and NaOH solutions and the expansibility of the ACR were studied by concrete microbar tests and expansion stress tests. Moreover, the crack characteristics and reaction products were systematically studied by XRD, Polarizing microscopy and SEM-EDS to explain the root cause of the expansion of dolomitic rocks.

## 2. Materials and Methods

### 2.1. Materials

#### 2.1.1. Rocks

**Figure 1:**
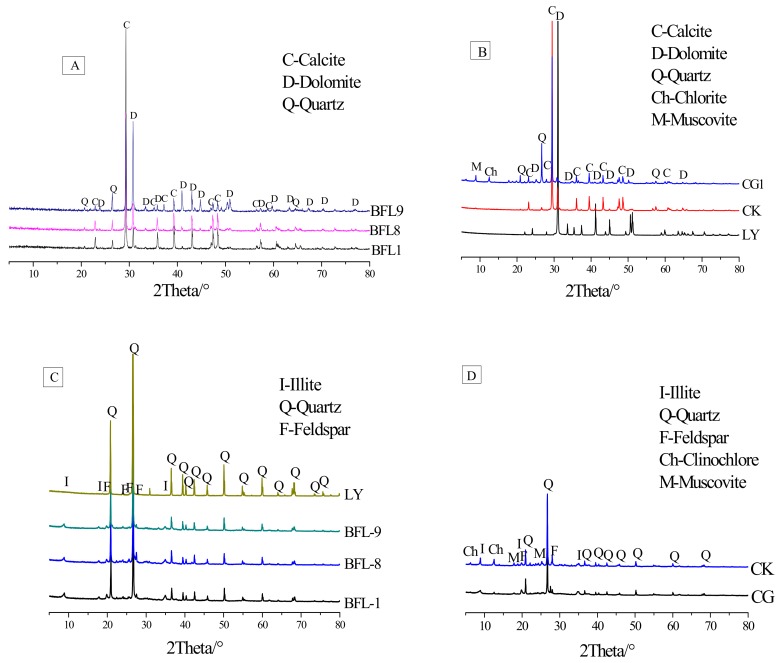
XRD patterns of rocks and the acid-insoluble residues of rocks: (**A**,**B**) Dolomitic rocks; (**C**,**D**) Acid-insoluble residues of dolomitic rocks.

Dolomitic rocks CK, CG1, BFL1, BFL8 BFL9 and LY were selected. Dolomitic limestone CK was obtained from Kingston (ON, Canada). Dolomitic rocks CG1, BFL1, BFL8, BFL-9 and LY were derived from China. Figure 1 shows the XRD pattern of the rocks and the acid-insoluble residues of rocks. The chemical composition of the dolomitic rocks is shown in Table 1.

It can be seen in Figure 1A,B that the dolomitic rocks BFL1, BFL8, BFL9, CG1 and CK contain calcite, dolomite and a small amount of quartz, there is also a small amount of chlorite and muscovite in CG1and CK. Dolomitic rock LY consist of dolomite. The acid-insoluble residues of dolomitic rocks BFL1, BFL8, BFL9, LY, CG1 and CK were mainly composed of quartz, illite and feldspar. In addition, a small amount of muscovite and clinochlore were contained in the acid-insoluble residues of CG and CK, as shown in Figure 1C,D. According to RILEMAAR-2 [20] standards, CG1 and CK rocks underwent the ASR, while BFL1, BFL8, BFL9 and LY were not affected by ASR activity.

#### 2.1.2. Cement and Chemical Agents

**Figure 2:**
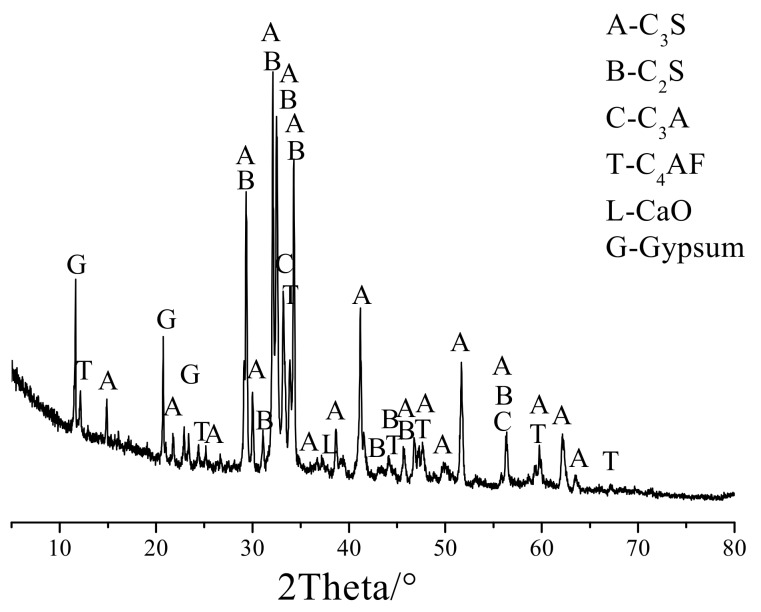
The XRD pattern of the homemade cement clinker.

Two kinds of cement were used in this experiment: ordinary Portland cement and a homemade cement without K^+^, Na^+^ and Mg^2+^. The ordinary Portland cement used was P·II 52.5 Portland cement produced by the Onoda Cement Company (Jiangnan, China). The alkali content (equivalent Na_2_Oeq) was 0.56%, and its chemical composition is shown in Table 2. The homemade cement clinker without K^+^, Na^+^ and Mg^2+^ was fired according to the calculated ratio. Table 3 shows the raw materials composition of the cement clinker without alkali. Finally, the cement clinker and gypsum were evenly mixed for 12 h at a weight of 1/19 to obtain homogeneous complete cement without K^+^, Na^+^ and Mg^2+^.The purpose of using cement without alkali was to eliminate expansion originating from the ASR. Figure 2 shows the XRD pattern of the cement without K^+^, Na^+^ and Mg^2+^. Additionally, Rietveld analysis was utilized to investigate the mineral contents of the cement clinker without K^+^, Na^+^ and Mg^2+^, as shown in Table 4.

Chemically pure NaOH agents were used to prepare 1 mol/L NaOH solutions, and commercial 25 wt% TMAH solutions were used to prepare 1 mol/L TMAH solutions. The chemical formula of TMAH is (CH_3_)_4_NOH, which is an organic alkali. The pH value of the 1 mol/L TMAH solution was higher than 13.

### 2.2. Methods

#### 2.2.1. Concrete Microbar Test

**Figure 3:**
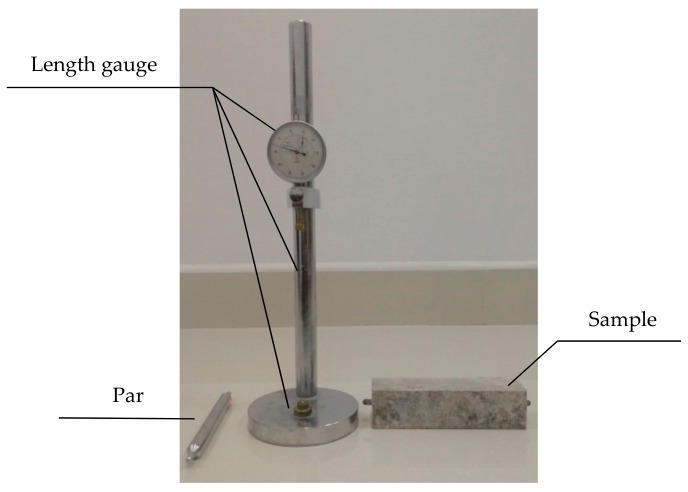
Length measuring instrument and test samples.

Concrete microbars (4 cm × 4 cm × 16 cm) were prepared with homemade cement and ordinary Portland cement as well as dolomitic rocks with grain sizes of 5–10 mm according to RILEM AAR-5 [21]. The alkali equivalent (equivalent Na_2_Oeq) of the cement was adjusted to 1.50 wt% by adding of 25 wt% TMAH and NaOH solution into the mixing water, the ratio of aggregate to cement was adjusted to 1/1, and the water-cement ratio was adjusted to 0.32. All specimens in moulds were placed in a moist environment (RH = 98%) at 20 °C. After 24 h, the specimens were removed from the moulds. Then measure the initial length of the sample and cured it in 1 mol/L NaOH and TMAH solutions. The length gauge and sample are shown in Figure 3. When curing to a set age, remove the sample and measure the length, then calculated the expansion rate according to Equation (1):

(1) $$P_{t}=\frac{L_{t}-L_{o}}{L_{o}-2b}\times 100\%$$

where P_t_ is the expansion rate after t days of curing, %; L_t_ is the test piece length after t days of curing, mm; L_0_ is the initial length of the test piece, mm; and b is the length of nail embedded in concrete, mm.

#### 2.2.2. Expansion Stress Test

**Figure 4:**
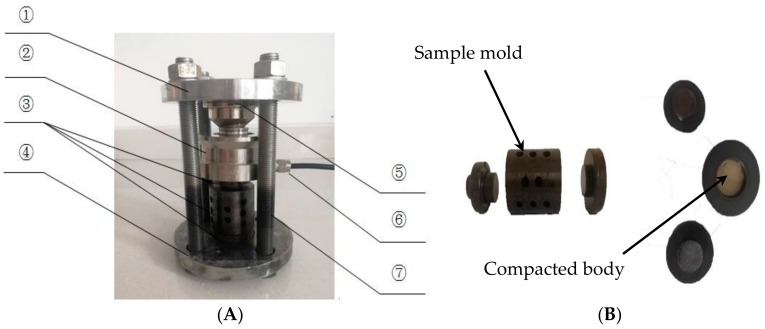
A schematic diagram of the expansion stress testing apparatus (①: top plate; ②: sensor; ③: sample mould; ④: bottom plate; ⑤: anti-load measuring head; ⑥: data acquisition system; ⑦: constrained screw). (**A**) Expansion stress test apparatus; (**B**) Sample mold.

In this study, an expansion stress test apparatus was used, the purpose of which was to investigate whether the alkali dolomite reaction can generate expansion stress. The stress apparatus is shown in Figure 4. Dolomite LY was crushed and finely ground to pass through an 80 micron sieve. Approximately 50 g of dolomite powder was put into the sample mould, and the parameters of the compacting machine (pressure 400 MPa; dwell time 5 s) were adjusted to prepare a dolomite powder compacted body. Then we put the mould into the expansion stress test apparatus, and the pressure was adjusted to 15 ± 0.1 MPa by tightening the nut. Finally, the test apparatus was placed in a 60 °C curing box containing a 1 mol/L NaOH or TMAH solutions, and the data were recorded. The expansion stress was calculated according to Equation (2):

(2) $$\sigma =\frac{{4(F}_{t}-F_{o})g}{{\pi d}^{2}}$$

where σ is the expansion stress (MPa); F_t_ is the sensor value at time t (kg); F_0_ is the initial value of the sensor (kg); g is the gravity acceleration, which has a value 9.8 m/s^2^; d is the inner diameter of the mould, which has a value of 24 mm; and π is 3.14. 

#### 2.2.3. Thin Section Petrography

The samples taken from the concrete microbar were cut into thin sections for optical microscopy to examine the presence of reaction products and expansion cracks originating from the aggregate. A polarizing optical microscope (Optiphot-II Pol reflecting light apparatus, ×25–400, Nikon, Tokyo, Japan) with transmitted light was used. The preparation of thin sections was performed according to the section “Thin section specimen preparation” [22].

#### 2.2.4. Laser Scanning Confocal Microscopy (LSCM)

The sample taken from the concrete microbar was polished, and the sample was observed by LSCM (TCS SP8 X, Leica, Wetzlar, Germany) to find the expansion source that caused the concrete to crack. Under the LSCM, the development of cracks in the aggregate and the products present can be clearly seen to determine the origin of the expansion.

#### 2.2.5. Analysis by SEM-EDS and X-ray Diffraction

Due to the complex composition of carbonate aggregates, which usually contain a certain amount of clay and microcrystalline quartz, SEM-EDS and X-ray diffraction analyses were used to examine the reaction products. The combination of SEM and element mapping is very effective in distinguishing product details in carbonate aggregates. Chemical element mapping (for Ca, Mg, Si, Na, K, etc.) of thin sections examined under a polarizing microscope was performed using EPMA/EDS to visualize the element distribution in the reacted aggregates. In addition, the fracture surface of carbonate aggregates from the concrete microbars was observed by SEM-EDS to identify the type of reaction products. The samples were characterized under an FE-SEM Ultra Plus microscope (Zeiss, Jena, Germany) equipped with EDS. X-ray diffraction (Smart Lab, Rigaku, Tokyo, Japan) was used to analyse the composition of the carbonate aggregate, the cement without alkali and the reaction products.

## 3. Results

### 3.1. Expansion of Concrete Microbars

**Figure 5:**
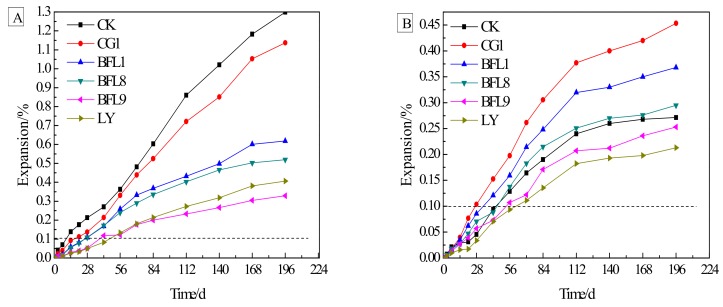
Expansions of concrete microbars: (**A**) concrete microbars cured in 1 mol/L NaOH at 80 °C; (**B**) concrete microbars cured in 1 mol/L TMAH at 80 °C.

Figure 5A,B show the expansion of concrete microbars cured at 80 °C in 1 mol/L NaOH and TMAH solutions, respectively. Figure 5A shows that the expansion of the concrete microbars prepared from dolomitic rocks and ordinary Portland cement in a NaOH solution is very obvious. The expansion rates of the concrete microbars prepared by CK, CG1, BFL1, BFL8, BFL9 and LY at age 28d were 0.21%, 0.13%, 0.11%, 0.10%, 0.05% and 0.02%, respectively. According to the RILEMAAR-5 [21] standards, CK, CG1, BFL1 and BFL8 were affected by ACR activity. As the age increased, the expansion gradually increases. At the later stage, the expansion rate of the concrete began to decrease and become stable. From Figure 5B, we can see that when the curing solution is TMAH, the expansion rate of the concrete microbars prepared from dolomitic rock and homemade cement without alkali is very slow before 28 d. The expansion of concrete microbars prepared from CK, CG1, BFL1, BFL8, BFL9 and LY at 14 d were 0.03%, 0.04%, 0.03%, 0.02%, 0.02% and 0.01%, respectively. After 28 d, the concrete had obviously expanded. As the age increased, the concrete expanded more. The expansion of the concrete microbars reached 0.27%, 0.45%, 0.36%, 0.29%, 0.25% and 0.21% at 196 d.

The slow expansion rate of the concrete microbars cured in TMAH solution at the early stage can be attributed to the shrinkage of cement without alkali. The shrinkage of cement will offset some of the concrete expansion caused by the ACR. As seen by comparing Figure 5A,B, the expansion of the concrete in the TMAH solution is less than the expansion of the concrete in the NaOH solution due to exclusion of the contribution of the ASR to the expansion. As Deng et al. [23] believed, the ASR may contribute to the expansion of concrete microbars cured in a 1 mol/L NaOH solution.

### 3.2. Characterization of Expansive Stress

**Figure 6:**
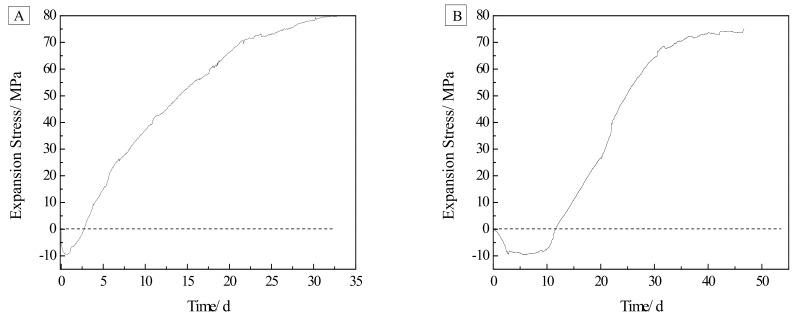
Expansion stress curve of compacted bodies: (**A**) compacted body cured in 1 mol/L NaOH solution at 60 °C; (**B**) compacted body cured in 1 mol/L TMAH solution at 60 °C.

Figure 6A shows the expansion stress curve of the dolomite powdered compacted body cured in 1 mol/L NaOH solution. The expansion stress continuously decreased in the early stage, and gradually increased after a short stationary period. After 25 days, the expansion stress growth rate decreased and gradually stabilized, and the 33 days expansion stress reached 81 MPa. Figure 6B shows the expansion stress curve of the dolomite compacted body cured in a 1 mol/L TMAH solution. As seen by comparing Figure 6A with Figure 6B, there was no significant difference in the development of the expansion stress of the compacted bodies in the NaOH solution and the TMAH solution. In the TMAH solution, the development of expansion stress was roughly divided into three stages, namely, the decline period, the stationary period and the growth period. During the descending period, the agglomeration of fine dolomite particles led to shrinkage of the dolomite compacted body, which led to a decrease in the expansive stress. In the stable period, the chemical reaction between the dolomite crystal and TMAH solution caused expansion of the dolomite compacted body, and the expansion of the compacted body just offset its volume shrinkage. As the reaction progresses, the expansion gradually increased, and the expansion stress of the dolomite compacted body began to increase. After 30 days, the growth rate decreased and tended to be stable, and the expansion stress reached 75 MPa on 45 days.

**Figure 7:**
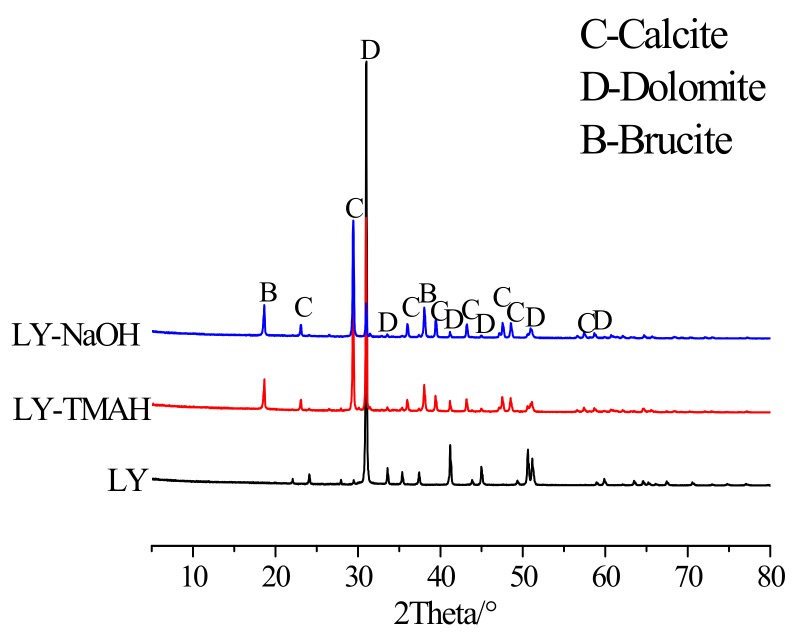
XRD patterns of untreated dolomite powdered and dolomite powdered compacted bodies in 1 mol/L NaOH and TMAH solutions at 60 °C for 55 days.

To determine how the expansion stress of the dolomite powdered compacted body was generated, X-ray diffraction analysis was performed on the untreated dolomite powder LY and the dolomite powdered compactted bodies cured in NaOH and TMAH solutions. The results of the XRD analysis are shown in Figure 7. It can be seen from Figure 7 that untreated dolomite powder is composed of dolomite, while large amounts of calcite and brucite were formed in the dolomite powdered compacted bodies in the NaOH and TMAH solutions. 

**Figure 8:**
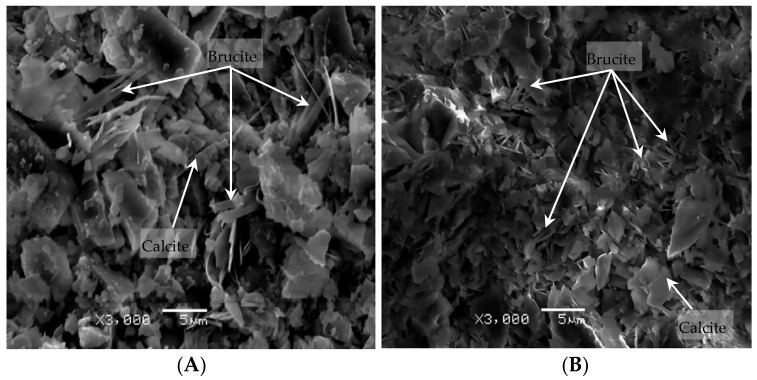
SEM images of reaction products of dolomite powdered compacted bodies: (**A**) Compacted body, NaOH 60 °C 45 days; (**B**) Compacted body, TMAH 60 °C 45 days.

Figure 8 shows the micro-morphology of the products in the dolomite compacted body after the reaction. It can be seen from Figure 8 that the dolomite powdered compacted bodies in the NaOH and TMAH solutions produced a large amount of flaky brucite and columnar or plate-shaped calcite. It can be inferred that the dolomite crystals in the dolomite powdered compacted body reacted with TMAH to form brucite and calcite. This chemical reaction is the same as the chemical reaction between dolomite crystals and NaOH solution, i.e., dedolomitization. This is consistent with the theory proposed by Hadley [5] that the dolomite will undergo dedolomitization in NaOH solution. Therefore, it can be considered that the ADR is the cause of the expansion stress of the dolomite compacted body. 

### 3.3. Optical Microscopy

#### 3.3.1. Polarizing Microscopy

**Figure 9:**
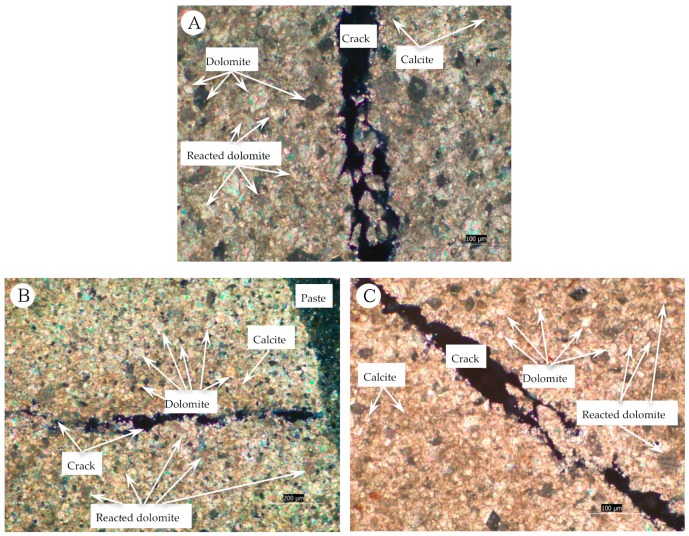
Thin section of concrete microbars prepared with dolomitic aggregate: (**A**) BFL1, NaOH 80 °C 196 days; (**B**) BFL8, NaOH 80 °C 196 days; (**C**) BFL1, TMAH 80 °C 196 days.

The thin sections of concrete microbars cured in NaOH and TMAH solutions were examined by polarizing microscopy. Figure 9A shows the crack characteristics of concrete prepared with BFL-1 aggregates in a NaOH solution. Figure 9A shows that a large number of rhombohedral dolomite crystals were dispersed in the calcite matrix and that severe cracking occurred in the dolomite-rich region. We can clearly see that there is no ASRgel filling in the cracks originating from the dolomite enrichment area. In addition, obvious dedolomitization occurred in the dolomite region, because the dolomite crystals exhibited dark to light brownish colour after the reaction [1],and the characteristics of the reaction were similar to those reported by Qian [24] and Prinčič [25]. Therefore, it can be inferred that the cause of cracking of the concrete microbars was related to dedolomitization. The cracking characteristics of concrete microbars prepared from BFL-8 in NaOH solution are shown in Figure 9B. Figure 9B shows that the rhombic dolomite crystals in the dolomite enrichment area are densely distributed in the calcite matrix. The crack originating from the dolomite region developed along the surface of the dolomite crystals and eventually penetrated the enrichment area into the cement paste. At the same time, no ASRgel was found in the crack and the dedolomitization occurred around the crack. Since there is a certain amount of microcrystalline quartz in BFL1 and BFL8, even if the expansion was caused by the ADR, it is difficult to prove that the ADR was the cause of concrete cracking because the contribution of the ASR to expansion cannot be avoided. To further determine the causes of concrete cracking, the concrete microbars cured in TMAH were cut into thin sections for polarizing microscopy observation. As shown in Figure 9C, the expansion cracks originate from the dolomite dispersed area, and the crack was empty. In addition, dedolomitization can also be seen in the dolomite areas. Therefore, it is speculated that dedolomitization may play a key role in the expansion of concrete microbars. 

#### 3.3.2. Laser Scanning Confocal Microscopy (LSCM)

**Figure 10:**
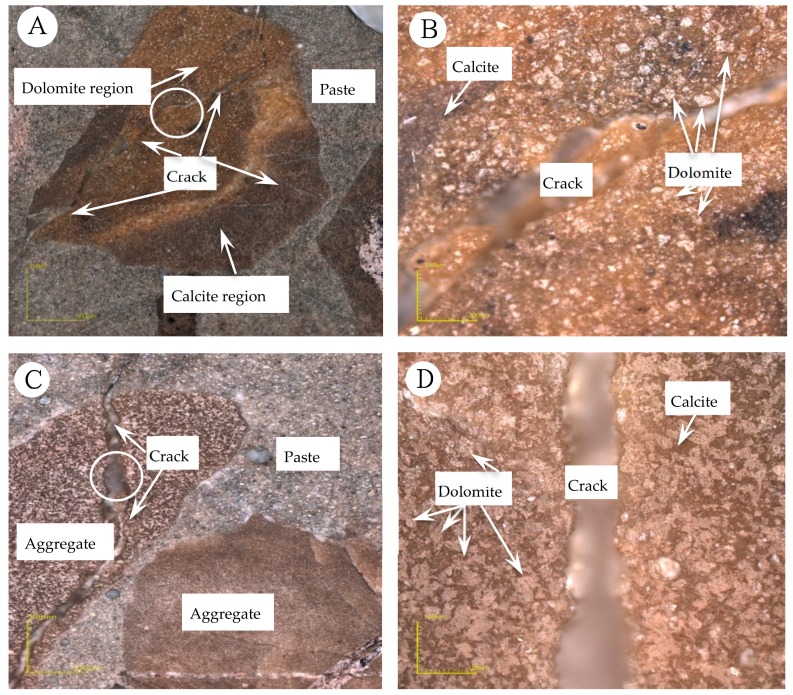
Expansion cracks of concrete microbars prepared with dolomitic aggregate: (**A**,**B**) BFL1, NaOH 80 °C 196 days; (**C**,**D**) BFL1, TMAH 80 °C 196 days.

Figure 10A,B show the expansion cracks of concrete microbar prepared with BFL1 aggregate cured in NaOH solution. Figure 10A shows that the rock aggregate was composed of dolomite rich area and calcite area and the boundary was clear. In the calcite region, micro-cracks can be seen, but in the dolomite-rich region, more severe expansion cracks were formed, which extend through the dolomite-rich region to the paste. In addition, it can be found that the cracks in the dolomite-rich region extend outwards from coarse to fine, indicating that the dolomite-rich region is likely to be the site of expansion initiation. On the other hand, it can be clearly seen that the crack was empty and there is no ASRgel filling (Figure 10B). Figure 10C,D show the expansion cracks of concrete microbar prepared with BFL1 aggregate cured in TMAH solution. It can be seen from Figure 9C that the crack development of the concrete microbar in the TMAH solution is similar to that in the NaOH solution (Figure 10A). The expansion crack originating from the dolomite-rich region passes through the aggregate and extends to the paste. The crack extends outward from coarse to fine and finally stays in the paste. Moreover, Figure 10D shows that the crack was empty and there is no filler. By using LSCM to analyze the cracks in the concrete microbars, it can be reasonably considered that the expansion source causing the expansion and cracking of the concrete is in the dolomite enrichment area.

### 3.4. Analysis by SEM and EDS

#### 3.4.1. Dolomitic Limestone BFL1, NaOH solution, 80 °C, 196 days

**Figure 11:**
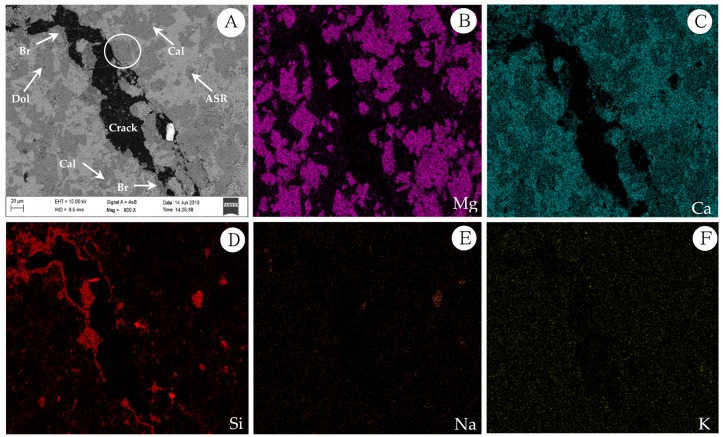
Element mapping of the RILEM AAR-5 concrete bars (**A**–**F**): Concrete microbars made with the dolomitic BFL1 aggregate in NaOH at 80 °C after 196 days. (**A**) SEM photograph; (**B**) Mg in the spotted brucite and dolomite; (**C**) Ca in the spotted calcite and dolomite; (**D**) Si images; (**E**) Na images; (**F**) K images.

**Figure 12:**
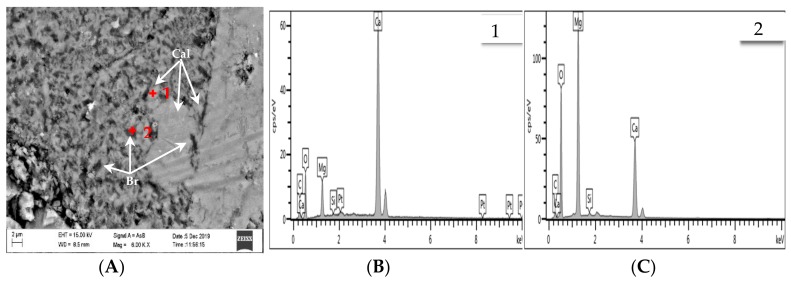
BSE and EDS images of products in concrete bars cured in 1 mol/L NaOH at 80 °C: (**A**) BSE image of the products; (**B**) EDS image of calcite; (**C**) EDS image of brucite.

**Figure 13:**
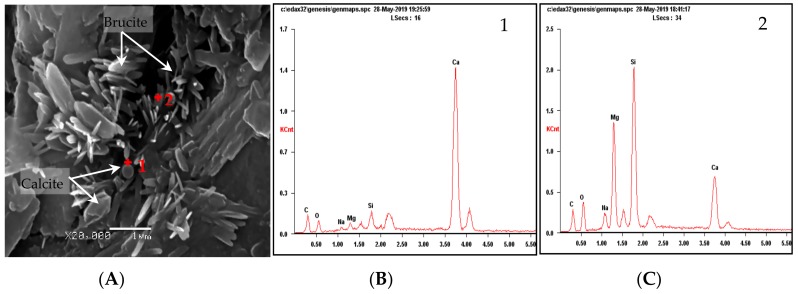
SEM and EDS images of products in concrete bars cured in 1 mol/L NaOH at 80 °C: (**A**) SEM image of the products; (**B**) EDS image of calcite; (**C**) EDS image of brucite.

Chemical element mapping of the polarizing thin sections was performed using EPMA/EDS to further clarify the main reason for the expansion of the concrete microbars. Some of the selected elements are shown in Figure 11 and Figure 12 shows a partially enlarged view of Figure 11A. The distribution of Mg and Ca elements indicates that the crack passes through the dolomite enrichment area (Figure 11B,C). However, the dedolomitization process in this region was not obvious, and the characteristic structure generated after dedolomitization was not observed. Similar results have been described in Fecteau’s research [26]. This difference is thought to be related to the type of rock. Dolostone has a distinct myrmekitic texture after dedolomitization, which has been reported [27,28,29]. However, we can still see spots of brucite and calcite formed by dedolomitization (Figure 11A and Figure 12). Based on the description in the literature, the dark spots correspond to brucite and the lighter spots correspond to calcite [12]. As shown in Figure 12, the dark spots proved to be brucite and the lighter spots were calcite. Furthermore, it can be seen from Figure 11A that there is no obvious ASRgel filled crack (width > 9 μm), which can be confirmed by the distribution of Si, Na and K elements (Figure 11D–F). The so-called ACR proposed by Katama [12] leads to concrete cracking that must be accompanied by ASRgel, which was not found in this study. However, the concrete microbars in the NaOH solution underwent the ASR, which can be proved by the distribution of Si and Na elements. On the other hand, the fracture surface of the concrete bars was observed by SEM for further identification of the reaction products. Based on the SEM image and EDS analysis, flake brucite and columnar calcite formed around the dolomite after the reaction (Figure 13), here, the reaction product of the ACR is similar to that reported by Du-You LU et al. [30]. Usually, the ASRgel found in the mortar is amorphous, and its crystalline phase, previously reported to be a Rosette structure, is not found here. These observations indicate that when the curing solution is NaOH, the ASR occurred and may be involved in the deterioration of concrete microbars, but the ACR was the main cause of concrete expansion and cracking.

#### 3.4.2. Dolomitic limestone BFL1, TMAH solution, 80 °C, 196 days

**Figure 14:**
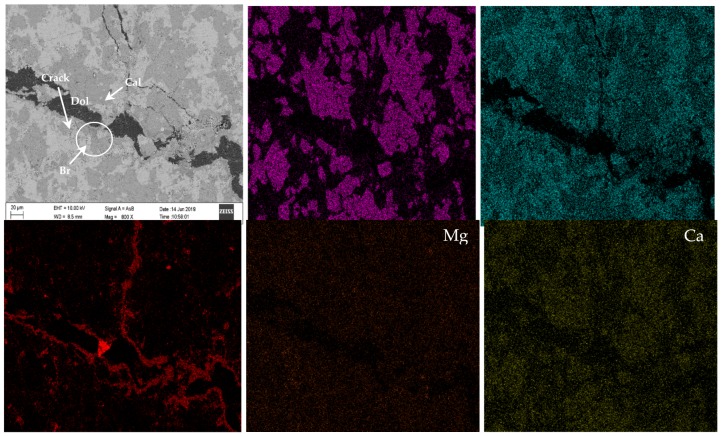
Element mapping of the RILEM AAR-5 concrete bars. (**A**–**F**): Concrete microbars made with the dolomitic BFL1 aggregate in TMAH at 80 °C after 196 d. (**A**) SEM photograph; (**B**) Mg in the spotted brucite and dolomite; (**C**) Ca in the spotted calcite and dolomite; (**D**) Si images; (**E**,**F**) Na, K images.

**Figure 15:**
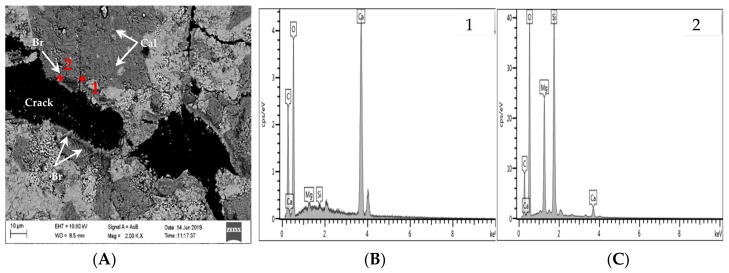
BSE and EDS images of products in concrete bars cured in 1 mol/L TMAH at 80 °C: (**A**) BSE image of the products; (**B**) EDS image of calcite; (**C**) EDS image of brucite.

**Figure 16:**
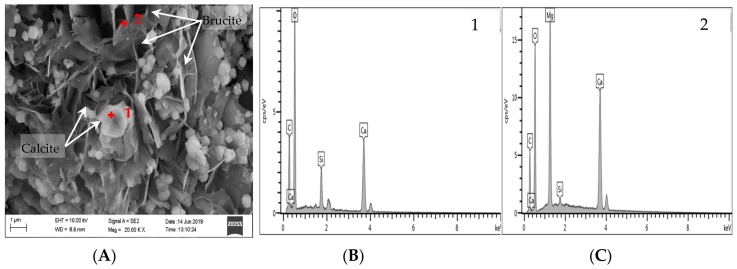
SEM and EDS images of products in concrete bars cured in 1 mol/L TMAH at 80 °C: (A) SEM image of the products; (B) EDS image of calcite; (C) EDS image of brucite.

The EDS analysis results of concrete microbars cured in TMAH are shown in Figure 14 and Figure 15, Figure 15 shows a partially enlarged view of Figure 12A. Obviously, no ASR occurred under the curing condition of the TMAH solution, and no ASRgel was found in the crack (Figure 14A and Figure 15). Si enrichment can be seen from the Si element distribution on both sides of the crack, which may be caused by the crack passing through the clay area, because the dolomitic limestone contains some clay, and the clay has a certain amount of Si (Figure 14D). According to the distribution of Mg and Ca elements in Figure 12B,C, it is easy to observe cracks in the dolomite enrichment area, and the ADR obviously occurred (Figure 15). The EDS analysis results showed that the dark spots were brucite and the lighter spots were calcite. These products were produced by the ACR, and the product morphology is shown in Figure 16. It can be seen from Figure 16 that flake brucite and columnar calcite were formed, and the reaction products of the ACR are consistent with the results of Tong and Zhang et al. [9,31]. Here, there is no ASR reaction product formation. The results of SEM-EDS analysis show that when the curing solution was TMAH, the dolomite crystals reacted with the TMAH solution to form brucite and calcite. This chemical reaction is the same as dedolomitization and will cause expansion and cracking of concrete microbars. 

## 4. Discussion

At present, the expansibility of the ACR is still a matter of debate. However, in some cases, the lack of detailed research could lead to misunderstandings and even incorrect conclusions. For this reason, we have conducted a detailed study on whether ACR can generate expansion.

From the results of the above expansion test, it is clear that the dolomitic rocks in this study exhibited obvious expansive behavior due to alkali attack. Actually, the reactions that occurring inside dolomitic rocks are very complicated [32], and the results obtained here have confirmed the expansibility of the ACR. However, this does not mean that other factors may not affect the expansion of the reactive carbonate rock. For example, the degree of dedolomitization, acid insoluble residues (such as clay and quartz) and rock texture, all contribute to the expansion of the active carbonate rock. Katayama [11] and Locati Francisco [27] study found that the degree of dedolomitization in the dolomite is proportional to the content of dolomite crystals. Nevertheless, the expansions of the concrete microbars tested were not related to the content of dolomite crystals in the rocks [27,33]. In this experiment, the dolomitic rock LY contains the most dolomite crystals (Table 1), but the expansion caused by it is small, which is the same as the experimental results of Deng et al. [34]. In addition, as shown in Figure 2, dolomitic rocks usually contain some acid-insoluble residues, and their contribution to expansion are difficult to evaluate, which complicates the interpretation of the ACR [35]. Usually clay and clay-like minerals provide channels for alkali to enter dolomite crystals and weaken the carbonate skeleton, thus promote expansion [8,16,26]. At the same time, many researchers have demonstrated that the ACR expansion behavior of aggregates is highly dependent on the textures of the aggregates [16,24,36]. Nevertheless, according to Qian et al. [24] and Tong [35], it is found that rock texture and clay are not always necessary for the ACR expansion process, but dedolomitization was essential. The above results indicate that whether the curing solution is NaOH or TMAH solution, the expansion difference between concrete microbars prepared from different rocks cannot be explained by a single factor, because the expansion process of the ACR is very complicated and requires detailed systematic research. On the other hand, because the dolomite powder compacted body is not affected by the rock texture and contains almost no clay (Figure 2), the expansion stress of the compacted body cured in the TMAH solution is directly generated by the dedolomitization (Figure 6, Figure 7 and Figure 8B), and the expansion corresponds to the degree of dedolomitization [32]. Compared with the expansion process of dolomite compacted body cured in NaOH solution, there is no obvious difference (Figure 6). This is because the dolomite LY contains only less than 0.2% of reactive quartz (Table 1), which can ignore the effect of the ASR on expansion [34,37], and quantitative analysis by XRD found that the degree of dedolomitization of the dolomite powder compacted body in the TMAH solution was 57.53%, while the dolomite powder compacted body in the NaOH solution was 61.99%.Therefore, it can be reasonably considered that the expansion of the compacted body cured in NaOH solution is mainly due to the ACR (Figure 7 and Figure 8A).

Dolomitic usually contains some quartz, but only certain components of these quartz are reactive, such as microcrystalline or cryptocrystalline quartz [38,39,40]. These reactive quartz can cause ASR with alkali, which is why some researchers suspect the existence of deleterious ACR [41]. However, in the reaction system of NaOH solution, no significant amount of ASRgel was found in the cracks originating from the dolomite-rich region (Figure 9, Figure 10 and Figure 11A), despite the use of polished thin sections prepared in accordance with the method used by Katayama [12]. Although no ASRgel was found, it is not possible to deny that the ASR may take a part in the expansive reaction, because the ASR reaction did occurred (Figure 11D,E). Moreover, there is a significant amount of brucite and calcite around the cracks (Figure 12 and Figure 13). These reaction products were all produced by de-dolomitization. These results indicate that the expansion of concrete cannot be attributed to the ASR, the ADR may play an important contributing role in the expansion of concrete. It can then be concluded that the alkali dolomite reaction is the root cause of the expansion. Similarly, no ASRgel was found in the concrete microbar cracks cured in the TMAH solution, and the ASR did not occurred (Figure 14). Only brucite and calcite, the products of the alkali dolomite reaction, were seen on both sides of the crack (Figure 15 and Figure 16). From the above results, it can be seen that in the TMAH reaction system, ACR is the only cause of concrete expansion. Obviously, ACR can cause concrete expansion, but more research is needed on how the alkali dolomite reaction process causes concrete expansion and cracking.

## 5. Conclusions

We compared the expansion behaviour of concrete and dolomite compacted bodies in TMAH solution and NaOH solution, and studied the expansion and cracking of the sample. From the physical measurement and microstructural analyses, the following major conclusions can be drawn.

Dolomite crystals in dolomitic rocks react with TMAH solution to form brucite and calcite. XRD and SEM-EDS analysis show that the chemical reaction between the dolomite crystal and TMAH solution was dedolomitization. In addition, the concrete microbars and dolomite powder compacts cured in the NaOH and TMAH solutions experienced obvious expansion, and the expansion stress of the dolomite powder compacts reached 81 MPa and 75 MPa at the end of the stress test. The expansion stress test and concrete microbar test results suggest that the ACR can produce expansion. Although both the ASR and the ACR were detected under the curing conditions of the NaOH solution, it was found by SEM-EDS observation that the cracks were mainly generated in the dolomite enrichment area and there was no ASRgel filling in the cracks, indicating that the expansion cracks were mainly caused by the ACR. In the TMAH reaction system, ASRgel was not seen in the cracks of the concrete microbars, and the ASR did not occur. At the same time, many ACR products were found around the crack, indicating that the expansion of the sample in the system is completely caused by the ACR.

## Figures and Tables

**Table 1:** Chemical composition of rocks (wt%).

Sample	Chemical Composition/%					
	Loss	SiO_2_	Fe_2_O_3_	Al_2_O_3_	CaO	MgO
BFL1	42.05	3.90	0.65	1.23	44.61	6.35
BFL8	42.06	2.68	0.26	0.93	48.65	4.39
BFL9	41.71	5.54	0.81	1.14	38.39	11.63
LY	47.05	0.20	0.16	0.18	28.68	20.36
CG1	32.01	18.42	2.14	5.00	34.07	4.84
CK	37.31	11.01	0.71	3.06	41.04	4.47

**Table 2:** Chemical composition of Portland cement (%).

LOI	SiO_2_	Fe_2_O_3_	Al_2_O_3_	CaO	MgO	K_2_O	Na_2_O	SO_3_	Total
2.91	19.33	2.86	4.83	64.10	2.25	0.67	0.12	2.58	99.65

**Table 3:** Raw material composition of cement clinker without alkali (wt%).

CaCO_3_	SiO_2_	Al_2_O_3_	Fe_2_O_3_	Total
78.18	14.03	4.40	3.39	100

**Table 4:** The mineral contents of cement clinker without alkali (%).

C_3_S	C_2_S	C_4_AF	C_3_A	f-CaO	f-MgO
63.50	12.40	10.60	13.50	0.10	0
